# Cocktail of chelated minerals and phytogenic feed additives in the poultry industry: A review

**DOI:** 10.14202/vetworld.2021.364-371

**Published:** 2021-02-09

**Authors:** Vishwanath Gopal Bhagwat, Ellusamy Balamurugan, Paramesh Rangesh

**Affiliations:** The Himalaya Drug Company, Makali, Bengaluru, Karnataka, India

**Keywords:** chelated, cocktail, inorganic, organic, phytogenic feed additives, poultry, production performance, supplement, trace minerals

## Abstract

This review article delineates the role of chelated minerals and phytogenic feed additives (PFAs) cocktail supplementation in improving the overall health status and production performance of poultry birds and its economic effects in the poultry industry. Organically complexed minerals have many advantages over inorganic sources. It has improved absorption and efficacy, which meets the bird’s requirements comfortably with a low-dose level. Hence, inorganic forms can be replaced with lower-dose levels of organic minerals without any adverse effects on production performances in broilers and layers. PFAs possess medicinal properties, such as antimicrobial, antioxidant, adaptogenic, and immunomodulatory, therefore, could be recommended as supplements. They are also growth promoters that enhance the overall health status and augment poultry birds’ production performance. Furthermore, the tremendous potential of PFAs could be extracted with the recent advances in science and technology. With the advantages of organically complexed minerals and multiple beneficial applications, there is a resurgence to develop PFAs as a cocktail of organic minerals to improve the overall health status of poultry birds and augment their productivity, which, in turn, helps the poultry industry to grow decisively and economically.

## Introduction

Minerals are vital for physical and mental well-being. They are components of all cells, including the blood, hormones, nerves, muscles, bones, teeth, and soft tissue. Some minerals are integral components of enzymes that catalyze biochemical reactions, including energy production, metabolism, nerve-impulse transmission, muscle contraction, and cell permeability. It is a well-known fact that in commercial poultry feed, the inorganic form of trace minerals is added (sulfate or oxide salts) at up to ten-fold higher dose than the National Research Council’s (NRC, USA) recommendations because of its low retention rates [1-3]. The excretion of unabsorbed inorganic trace minerals leads to environmental contamination [4]. Organic trace minerals have higher bioavailability than inorganic trace minerals. Hence, supplementation of organically complexed/chelated trace minerals could help avoid the use of higher dosages of inorganic trace minerals in poultry feed and could prevent environmental contamination because they have lower inclusion rates and reduced excretion [5,6].

Chelated complexes of minerals contain a central atom along with a ligand (proteins, carbohydrates, lipids, or amino acids) containing a minimum of one ligand atom (sulfur, oxygen, or nitrogen) with a pair of free electrons. The ligand atom is bound with the metal atom by a coordinate bond by donating an electron pair from the ligand to the electron acceptor [7].

**Figure-1:**
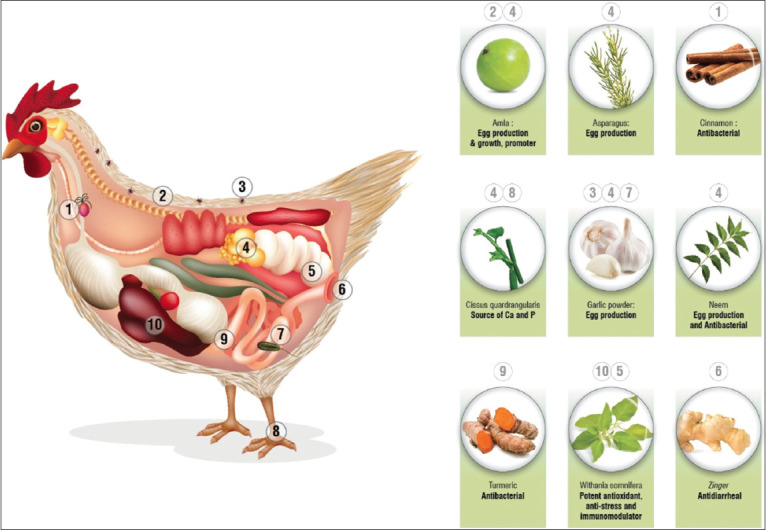
The beneficial application of herbs in poultry health and production. Source: Sheoran [8].

The inclusion of low organic mineral levels has been widely practiced due to their physiologic contributions (Figure-1) [8].

There are two main disadvantages of using inorganic trace minerals in the poultry diet. First, the contamination factor: Copper sulfate and zinc oxide are common inorganic sources of copper and zinc used in the poultry diet, but these two sources are derived from the steel industry and have a large number of contaminants, such as fluorine and cadmium, which get transferred into the poultry diet [9]. Second, the antagonistic effects between inorganic trace minerals could decline its metabolism and absorption rate. Chelate complexes of metals with amino acids are inert because of the ionic and covalent bonding between the ligand and mineral. Thus, these forms remain unaffected by the factors that lead to precipitation, similar to how it happens to inorganic minerals after the solubilization of salt [6]. Furthermore, the size and stability of improvement due to chelation of trace minerals could protect the trace minerals, while passing through the gastrointestinal tract and are absorbed in its intact form, without any degeneration of the amino acids [5].

The herbs and plant extracts used in animal feed are known as phytogenic feed additives (PFAs). They are of plant origin and are added to animal feed to enhance productivity by improving the digestibility, nutrient absorption, and elimination of pathogen residents in the animal gut [10-12]. Unlike ruminants, poultry does not have a natural bacterial flora capable of degrading all nutrients. Hence, the administration of antibiotic growth promoters (AGPs) is recommended to augment production performance and improve poultry birds’ state of health as they have limited immunity against infection due to colonization by pathogenic microorganisms. However, the WHO observed that it is impossible to use AGPs that cause antimicrobial resistance in animals. The European Union banned the systemic use of AGPs in animal feed at the beginning of 2006. The removal of AGPs from the poultry diet has detrimental effects on production performance. Moreover, the removal of AGPs increases the regeneration of pathogens, leading to the decline of poultry animals’ health status and causing economic losses to the poultry industry. Here, the addition of PFAs to poultry feed is suggested to improve the health status of animals and augment production performance [13].

This review article delineates the role of supplementing a cocktail of chelated minerals and PFAs in improving poultry birds’ overall health status and productivity performance and its economic effects in the poultry industry.

## Mechanism of Absorption of Organic Minerals

**Figure-2:**
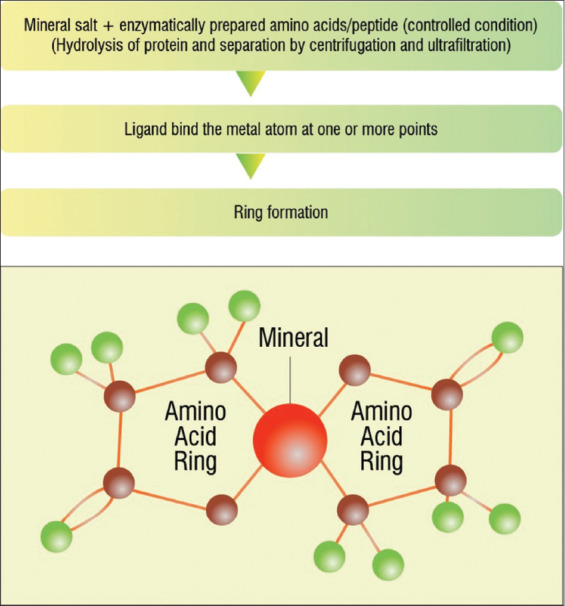
Chelated Mineral Preparation Techniques. Source: Sheoran [8].

After the intake of organic minerals from the poultry diet, mineral absorption occurs in any intestine region; however, metals are absorbed in the duodenum. The inert mineral complexes undergo hydrolysis in the stomach and enter the intestine lumen, where the ligand–mineral bonding occurs. Transporter ligand safeguards mineral interactions with dietary antagonists, such as oxalic acid, phytic acid, and antinutritional factor gossypol. In the intestine, the ligand–mineral complexes become absorbed through cells of the intestinal lining. Inorganic minerals get excreted in the feces due to the non-availability of an inorganic metal transporter (Figure-2) [8].

### Importance of Using Organic Trace Minerals in Poultry

Literature reports that the addition of chelated trace minerals in the poultry diet improves the performance parameters, bird health, and meat-quality traits. The performance of laying hen and eggshell quality improved following copper methionine supplementation compared with that of birds supplemented with copper sulfate [14]. Among chicks supplemented with chelated trace minerals, there was a significant increase in body weight gain, deposition of minerals in the tissue, and immunity and improved feed conversion ratio (FCR) compared with that among chicks supplemented with inorganic trace minerals at similar dose [15]. Broiler birds fed with a diet supplemented with organic chromium (0.5 ppm) showed increased body weight gain compared with birds supplemented with inorganic chromium sources [16]. There was an improved FCR in chicks fed a diet supplemented with organic sources of minerals compared with those fed with inorganic sources (1.63 vs. 1.74) [14]. In broiler fed mineral proteinate, ascites reduced from 5% to 2% [17]. Zinc/manganese methionine enhanced humoral and cell-mediated immune functions [18]. Hence, the chelated form is advantageous than the inorganic form.

Various research studies demonstrate that a dose of chelated minerals can be reduced in commercial broiler feed formulations without any negative effects on their production performance [5,19-21], antioxidant defense systems [22], hematological and biochemical parameters, and meat quality parameters [23]. Administration of chelated minerals at 25% of the NRC’s standard recommendations has no detrimental effect on performance parameters, such as body weight gain and feed intake [24]. Supplementation of chelated trace minerals at 20% of inorganic trace minerals has no negative effect on body weight gain and FCR, with an advantage of a reduction in environmental contamination due to the lower excretion of minerals [4]. Furthermore, literature reports revealed that the replacement of inorganic trace minerals with chelated minerals results in the augmentation of immunity parameters in chicks [25].

### The Effect of PFAs in Poultry

The poultry industry has been the principal source to meet animal protein’s global requirement among all other classes of livestock [26]. This can only be further strengthened by controlling infectious diseases as well as growth and production disturbances that would otherwise impose severe economic losses to the poultry sector. The emergence of new pathogens or variants of old pathogenic microorganisms can spread rapidly and affect the entire flock [26,27].

Since ancient times, plants and plant parts have a pivotal role as medicine sources for indigenous poultry production systems. The existing indigenous technical knowledge inherited from the past generations has sustained the local poultry production system [28,29]. Various researchers have explored indigenous medicinal herbs and plant extracts (e.g., garlic, cinnamon, tulasi, ginger, yucca, turmeric, neem, thyme, rosemary, and lemon) to enhance poultry health and augment the production performance with favorable results [30-35]. Moreover, a literature survey showed that dietary supplementation with herbal preparations positively affects performance parameters, such as body weight gain, feed intake, feed efficiency and carcass traits, biochemical parameters, and immune responses among poultry birds [36].

## Herbs as Growth Promoters for Poultry

At present, there are many restrictions and bans on the use of various antibiotics and other medicinal products in the poultry diet because of the resultant bacterial resistance and possible transmission of antibiotic residues into the human food chain, which has been the utmost concern of poultry production [37,38]. In addition, the poultry feed industry is also confronting tremendous pressure from consumers to reduce AGPs addition in the poultry diet. A literature survey revealed that medicinal plants, plant extracts, and essential oils as PFAs in commercial broiler diet have beneficial effects on broiler performance parameters [39-42]. Furthermore, several authors have demonstrated that plant extracts and various phytobiotics originating from leaves, roots, tubers, herb fruit, spices, and other plants have shown the potential to enhance the production performances of poultry birds and thereby have proven their pivotal roles in strengthening the poultry industry [43,44]. These PFAs growth-enhancing effects could be attributed to the synergistic action of containing various active molecules and their potential to improve feed utilization efficiency [45].

## Herbs as Poultry Antioxidants

At present, the preference for natural antioxidants in food has increased due to health benefits, such as preventing oxidative stress and diseases. Lipid peroxidation susceptibility of poultry meat due to polyunsaturated fatty acids drawback creates a huge demand for plant-based antioxidants [46]. Various plants possess excellent antioxidant properties, for example, rosemary (*Rosmarinus officinalis*), olive leaves (*Olea europaea* L.), garden thyme (*Thymus vulgaris*), marjoram (*Origanum majorana*), sage (*Salvia officinalis*), and oregano (*Origanum vulgare*) [47-51]. Furthermore, it has been demonstrated that tulasi (*Ocimum sanctum*) and Ashwagandha (*Withania somnifera*) possess excellent adaptogenic and antistress properties [52]. Apart from these, spices such as cinnamon, peppermint, marjoram, wild marjoram, cloves, caraway, and nutmeg have antioxidant properties as they contain compounds, such as polyphenols, flavonoids, and terpenoids [50-53].

## Effects of Herbs on Enzymes in Poultry

Literature shows that supplementation of plant and plant extracts, as well as poultry diet, exerts positive and favorable influences on serum enzymes. Deshpande reported that tulasi leaf powder’s dietary supplementation causes a significant increase in serum cholesterol and high-density lipoprotein levels in laying hens [54]. The addition of turmeric rhizome powder to the broiler diet considerably reduces liver enzyme levels, such as alkaline transaminase (ALT) and alkaline phosphatase (ALP) [55]. In contrast, the addition of tulasi leaf powder ameliorates the lead-induced toxicity in cockerels by reducing liver enzyme levels [56]. Feeding of tulasi leaf powder to broilers neutralizes aflatoxins toxic effects by significantly decreasing the enzyme activities of aspartate transaminase, ALT, and ALP [57]. Lanjewar *et al*. [58] reported that supplementation of tulasi leaf powder in broiler diet caused a significant reduction in serum low-density lipoprotein cholesterol, total cholesterol, and triglycerides.

Furthermore, Gupta and Charan [59] reported that tulasi supplementation reduces serum glutamic oxaloacetic transaminase (SGOT) levels in broilers. In contrast, it has no significant effect on serum glutamic pyruvic transaminase (SGPT), creatinine, and uric acid levels. Furthermore, supplementation with herbal growth promoters, such as amla to broilers, reduces cholesterol level, increases serum ALP, and SGPT levels and has no effect on SGOT levels [60]. Furthermore, feeding broilers with tulasi leaf powder (0.5%) and selenium (0.3 ppm) significantly decreased lipid peroxidation and increased plasma glutathione levels [61].

**Figure-3:**
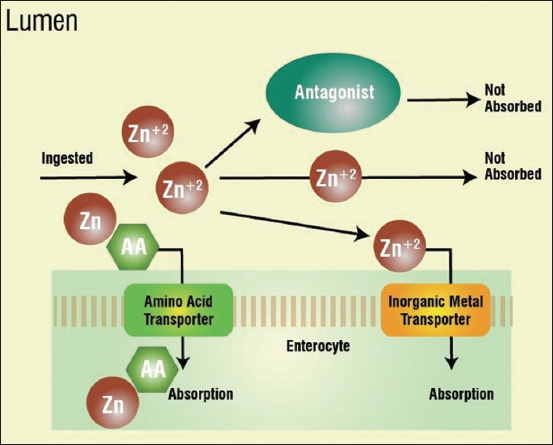
The mechanism of absorption of organic minerals. Source: Sheoran [8].

The beneficial applications, an overview of the usage of herbs, their modes of action for protecting poultry’s health, and the production performance-enhancing effects are diagrammatically represented in Figure-3.

## Synergistic and Antagonistic Effects of PFAs in Poultry Industry

The synergistic and antagonistic effects PFAs have been supported by various research studies reported in literature; Ren *et al*. [62] reported the synergistic beneficial impact of probiotics and phytobiotics. This study’s findings revealed that combinations of probiotics and phytobiotics lead to a more enhanced functionality than their individual supplementation by reducing the survival of potentially problematic bacteria, such as ESBL-producing *Escherichia coli* on intestinal bacterial composition and their metabolic activity in young broilers [62]. Phytogenic compounds represent a promising alternative to antibiotics because they consist of many active ingredients. Upadhaya and Kim revealed that minor components present are critical to the PFA activity and may have a synergistic influence [63]. Several other researchers reported that polyphenol, flavonoids, and carotenoids present in herbs play a crucial role in PFAs synergistic effects by stimulating increased digestion in birds, resulting in the birds’ enhanced growth [64-66]. Furthermore, literature reports reveal the synergistic and additive effects of the various molecules of the essential oils and their monoterpenoid components [67,68].

Literature reports also revealed that the antagonistic effect has been attributed to the interaction between non-oxygenated and oxygenated monoterpene hydrocarbons [69,70]. Essential oils possessing antimicrobial activities are mainly due to the presence of oxygenated terpenoids, while some hydrocarbons also exhibit antimicrobial effects. The interactions between these components may lead to antagonistic effects [71-73]. A mixture of essential oils interacts with each other, acting as antagonistic effects. The synergistic and antagonistic properties of PFAs are summarized in Table-1 [74-81].

**Table-1:** The Summary of synergistic and antagonistic properties of phytogenic feed additives.

Phytogenic feed additives	Effects	Reference
Thymol/carvacrol	Synergistic	Pei *et al*., [74]
Thymol/eugenol	Synergistic	Pei *et al*, [74]
Carvacrol/eugenol	Antagonistic	Gallucci *et al*., [75]
Carvacrol/myrcene	Antagonistic	Gallucci *et al*., [75]
Cinnamaldehyde/Thymol	Synergistic	Pei *et al*., [74]
Limonene/1,8-cineole	Synergistic	Vuuren and Viljoen [76]
α-pinene/Limonene	Synergistic	Tserennadmid *et al*., [77]
*Origanum vulgare/Rosmarinus Officinalis*	Synergistic	de Azeredo *et al*., [78]
*Lippia multiflora/Mentha piperita*	Synergistic	Bassolé *et al*, [79]
*Syzygium aromaticum/Rosmarinus officinalis*	Antagonistic	Fu *et al*, [80]
	Synergistic	
*Cymbopogon citratus/ Cymbopogon giganteus*	Synergistic	Bassolé *et al*., [81]

## Economic Importance of PFAs in Poultry Industry

Literature reveals that supplementation of PFAs is economical to the poultry industry. Various researchers report that the addition of turmeric powder at 0.5% in broiler diets significantly decreases the feed cost per unit live body weight gain (by 13.5% [82], 11.8% [83], and 6.2% [84] compared with the respective control diets). The addition of a combination of *Aloe vera* and *Curcuma longa* in broiler diet caused a significant difference in the feed cost per unit live body weight [85]. Furthermore, supplementation of a combination of amla, tulasi, and turmeric at a 0.25% dose along with a commercial broiler diet caused a 4% reduction in feed cost per unit live body weight gain in broilers [86].

To summarize, organically complexed minerals have many advantages over inorganic sources, such as improved absorption and efficacy that meet the bird’s requirements comfortably with low-dose levels. Consequently, it can be recommended that low dietary levels of organic minerals be replaced with inorganic forms without any negative influence on broilers’ growth performance and egg production in layers.

Medicinal herbs have proven to be valuable therapeutic aids for humans, animals, and birds since times immemorial. These herbs provide therapeutic relief and are often a safe alternative to conventional treatment and for boosting immune functions. Herbs can also effectively complement conventional medicines in disease management. The global trend in the poultry industry reveals a huge demand for poultry products. Thus, the poultry industry is facing production and bird health-related challenges. For a safe, sustainable, and profitable scenario, alternative remedies to conventional treatments have to be chosen. The practical implementations of PFAs are possible with the recent advancements in science and technology.

## Conclusion

Since organically complexed minerals have many advantages over inorganic sources, PFAs have multiple beneficial applications, and there is a resurgence to develop a cocktail of organic minerals and PFAs to improve the overall health status of birds and augment poultry birds’ productivity, which, in turn, helps the poultry industry to grow decisively and economically.

## Authors’ Contributions

VGB contributed to the original draft and conception of the specific review. EB contributed to the review, editing, and supported in supervision. VGB and PR worked on the final approval of the version to be published. All authors read and approved the final manuscript.
